# Active Sensor for Microwave Tissue Imaging with Bias-Switched Arrays

**DOI:** 10.3390/s18051447

**Published:** 2018-05-06

**Authors:** Farzad Foroutan, Natalia K. Nikolova

**Affiliations:** Department of Electrical and Computer Engineering, McMaster University, Hamilton, ON L8S 4L8, Canada; nikolova@ieee.org

**Keywords:** Microwave imaging, Ultra-wide band, active radio sensor, biased-switched array

## Abstract

A prototype of a bias-switched active sensor was developed and measured to establish the achievable dynamic range in a new generation of active arrays for microwave tissue imaging. The sensor integrates a printed slot antenna, a low-noise amplifier (LNA) and an active mixer in a single unit, which is sufficiently small to enable inter-sensor separation distance as small as 12 mm. The sensor’s input covers the bandwidth from 3 GHz to 7.5 GHz. Its output intermediate frequency (IF) is 30 MHz. The sensor is controlled by a simple bias-switching circuit, which switches ON and OFF the bias of the LNA and the mixer simultaneously. It was demonstrated experimentally that the dynamic range of the sensor, as determined by its ON and OFF states, is 109 dB and 118 dB at resolution bandwidths of 1 kHz and 100 Hz, respectively.

## 1. Introduction

Microwave imaging for biomedical applications has gained much attention in the last two decades. The relatively long wavelength allows for penetration in living tissue as deep as ten centimeters, especially at the low-gigahertz range. This makes microwave technology a promising candidate in diagnostic applications such as breast-cancer screening [1,2,3,4,5], bone-disease monitoring [6,7], brain-stroke [8,9,10] and lung-cancer diagnostics [11,12].

Currently, the main effort is in improving the image resolution and the diagnostic performance of the modality. There are several inherent challenges associated with this effort, which have so far hindered deployment in clinical practice. These include the complex pattern of the microwave propagation through the tissue [1,13], the wide statistical spread in the (complex) permittivity values of healthy tissues, which may overlap those of malignant tissues (e.g., in the breast), the uncertainties in the measured tissue dielectric properties, as well as the strong dissipation [1,14,15,16,17]. Many of these challenges are addressed through improvements in the reconstruction algorithms; however, the improvement of the hardware and the data quality is critically important.

This work focused on hardware development, which promises substantial improvement of the signal-to-noise ratio (SNR) and the dynamic range of the microwave acquisition system. With the advent of on-chip radio components such as low-noise amplifiers (LNAs) and mixers, it is now possible to integrate these with the antenna in a single active radio sensor whose output is at an intermediate frequency (IF) in the low megahertz range. This makes the sensor multiplexing simpler and cheaper since it circumvents the need for radio-frequency (RF) switches, transmission lines and connectors, which introduce loss and signal distortion. These RF components are also sensitive to operational factors such as temperature, bending, twisting, and poor contact, which lead to phase errors, measurement uncertainties and problematic trouble-shooting.

With the sensor’s output being at IF, it is possible to multiplex hundreds of sensors to a common IF port, where the signal can be further processed to extract magnitude and phase information. At IF, the required transmission lines are electrically very short, the losses are low and the impedance matching is simpler. This is a significant advantage over the existing RF-switched arrays. To our knowledge, the largest RF-switched antenna array for tissue imaging is the one reported by Klemm et al. [18], where 60 antennas are arranged on a hemispherical surface. In such arrays, each antenna connects to a large RF switching network through a high-quality RF cable and connectors. Careful calibration is required to account for the impact of the impedance match, the losses and the phase delays of all RF components.

When the number of array elements and their spacing do not provide sufficient spatial sampling, the RF-switched arrays are combined with mechanical scanning. For example, in [19], a hand-held compact impulse-radar detector is developed using 4×4 cross-shaped dome antenna array. Two single-pole-eight-throw (SP8T) RF switches are used to control the antenna array element in RF frequency. To overcome the drawback of the limited number of the elements, this prototype also employs rotation in order to obtain sufficient spatial sampling. This increases the scanning time and may lead to positioning uncertainties.

It should be noted that RF-switched antenna arrays offer significant improvement over the mechanically scanned systems (see, e.g., [20,21]), where positioning uncertainties and long acquisition times impede the implementation in clinical practice. Nonetheless, the number of elements that RF switches can multiplex remains limited.

To overcome the drawbacks of RF-switched arrays, the modulated scatterer technique (MST) has been proposed [22,23,24,25,26]. With this technique, the modulated scatterers are connected wirelessly to a single RF port, making the system flexible and capable of handling many sensing elements. However, these systems have relatively small dynamic range and narrow bandwidths.

The multiplexing of the active sensors in the imaging array can be conveniently carried out by switching on and off the bias of its active circuits (the LNA, the mixer and/or the IF amplifier). This is realized using a digital network, the design and fabrication of which is simple compared to an RF distribution network.

Table 1 provides a summary of the advantages of the bias-switched active-sensor array in comparison with the RF-switched, MST and scanned antenna arrays.

It should be noted that the MST arrays can be also viewed as RF-to-low-frequency converters.

The limiting factor in the electronic switching of a frequency-sweep sensor such as ours is the settling time in the receiver’s local oscillator. This settling time is particularly stringent in a coherent receiver, which employs a phase-locking loop, as explained in [27]. Depending on the IF bandwidth, the settling time ranges from tens of microseconds to tens of milliseconds [28,29], which sets the lower limit on the time required to perform the measurement at one frequency before moving to the next. In our bias-switched array, the bias is switched ON one element at a time. Once the bias for a given array element is ON, the frequency sweep is performed. Therefore, the period during which the bias of an element is ON equals $N_{\omega }t_{\mathrm{settle}}$, where $N_{\omega }$ is the number of frequency samples and $t_{\mathrm{settle}}$ is the settle time of the local oscillator. Equivalently, the rate at which the bias is switched is $N_{\omega }$ times lower than the rate at which the frequency hopping is performed.

The proposed sensor is suitable for applications in various configurations including planar, spherical and cylindrical surfaces. This is because the antennas are very small and can be easily deployed on curved surfaces. This is evident from the fact that the original antenna design has been used in a hemispherical array [19] which employs RF switching.

Here, the first application of the radio sensor is in a planar scenario. A planar microwave breast imaging system has already been reported in [30]. There, it is recognized that the planar configuration has certain advantages such as the fact that it is independent of the organ size. Furthermore, it is pointed out that the signal paths are shorter than those in hemi-spherical or cylindrical breast imaging, leading to better signal quality. At the same time, the compression is less than that in mammography.

A bias-switched UWB radio sensor for tissue imaging was first proposed in [31,32], where mixed-signal simulations suggested that a dynamic range as high as 127 dB can be achieved provided the IF receiver noise floor is sufficiently low, i.e., −126 dBm at a resolution bandwidth of 1 Hz. Here, we show the experimental results with a prototype of the radio sensor, which demonstrate a dynamic range of 118 dB when the resolution bandwidth is set at 100 Hz.

In Section 2, the design of the antenna array is discussed along with the tissue phantoms used in the experiments. Section 3 discusses the integration of the antenna array with the LNA and the mixer. Section 4 describes the measurements of the dynamic range of the active sensor. Discussion and conclusions are provided in Section 5 and Section 6, respectively.

## 2. Antenna Array Design

### 2.1. Carbon–Rubber Tissue Phantoms

Our phantom materials are custom-made to have complex permittivity similar to the weighted-average complex permittivity of breast tissue classified as *scattered fibroglandular*. This is a category under the classification of the American College of Radiology (ACR) Breast Imaging Reporting and Database System (BI-RADS^®^) that describes the “visually estimated content of fibroglandular-density tissue within the breasts” [33]. This classification pertains to mammography but is also used in microwave imaging since the breast density has direct implications for the complex permittivity values of the tissues and their distribution [34]. A breast categorized as *scattered fibroglandular* may have a few or moderate scattered areas of fibroglandular tissue.

The weighting of the tissue permittivity values is done according to their volume proportion in the breast [35]. The volume proportions are calculated according to magnetic-resonance (MR) images obtained at the Imaging Research Centre of St. Joseph’s Healthcare Hamilton (Imaging Research Centre (IRC), Brain Body Institute, St. Joseph Healthcare Hospital, 50 Charlton Ave. East, Hamilton, Ontario, courtesy of Drs. Michael Noseworthy and Colm Boylan) with permission from the Hamilton Research Ethics Board.

**Figure 1:**
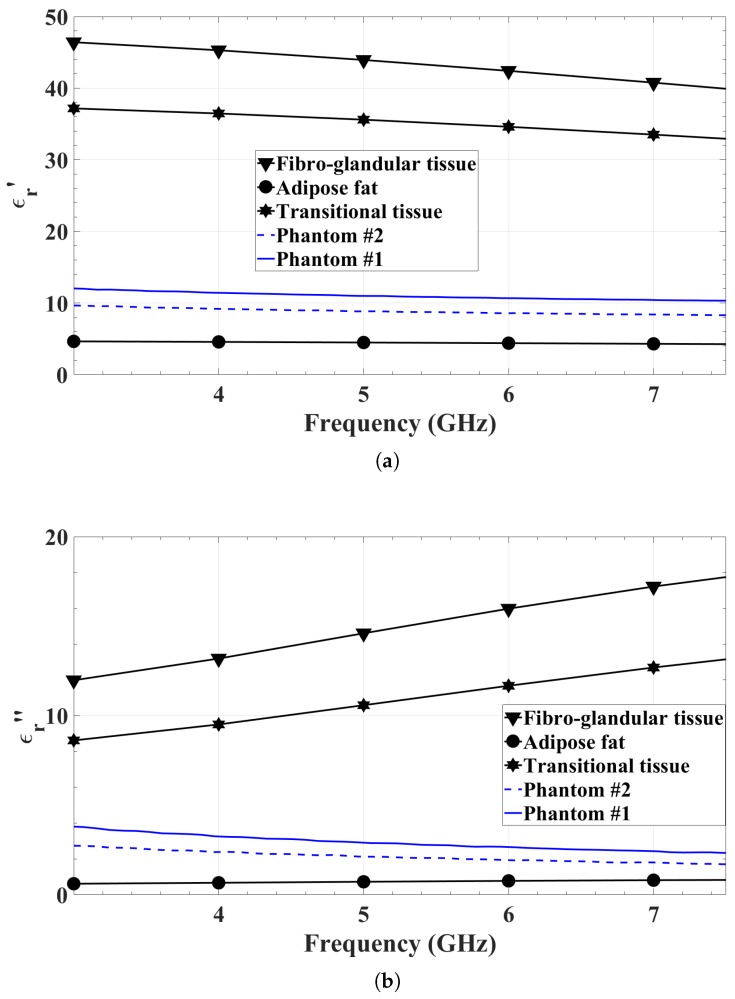
The measured relative permittivities of the two breast-tissue phantoms and the one-pole Debye models of the three healthy tissue types used in the calculation of the weighted-average permittivity of a scattered fibroglandular breast: (**a**) real part; and (**b**) imaginary part.

The tissue phantoms are made of carbon–polyurethane slabs whose dielectric properties are shown in Figure 1. The material is similar to that reported in [36].

In calculating the desired permittivity of the tissue phantoms, the permittivities of three healthy breast tissues have been used: (i) adipose fat; (ii) transitional tissue; and (iii) fibroglandular tissue. These values conform to results reported in [14,15,16,17]. They are computed using the one-pole Debye models suggested in [37]. The respective curves (taken from [35,38]) are plotted in Figure 1.

### 2.2. Antenna Array Element

The optimal choice of the frequency band for microwave tissue imaging is still a matter of investigation; however, most of the systems that have performed successful medical trials [18,19] use frequencies between 3 GHz and 8 GHz. This is because they employ direct inversion methods (e.g., delay-and-sum synthetic focusing), which are diffraction limited, i.e., their spatial resolution depends critically on the wavelength. As a result of these limitations, the higher the frequency is, the better the quality of the image. Thus, the choice of the frequency band is a compromise between penetration, which is better at low frequencies, and spatial resolution which is better at higher frequencies.

The sensor’s antenna must fulfill a number of design requirements. First, it must be able to operate in direct contact with the body surface without the need for submersion in coupling liquids. Second, it must be light-weight and allow for fabrication on a printed circuit board (PCB). This requirement stems from the need to integrate each antenna with a dedicated radio, which is central to the envisioned array concept. Third, the sensor must be able to take advantage of the ultra-wide bandwidth (UWB) of spectrum allocated by the Federal Communications Commission (FCC) between 3.1 GHz and 10.6 GHz. The goal here is to have an antenna that has satisfactory impedance match (i.e., reflection loss greater than 6 dB) from 3 GHz to at least 7 GHz. Fourth, for best efficiency, the antenna should not employ resistive coating or absorbing materials. Fifth, the antenna must be shielded from the back to reduce interference and to ensure maximum power in the forward direction toward the tissue.

Finally, the array must allow for user-defined spatial sampling rates and patterns (rectangular, circular, spiral, random, etc.). Thus, the center-to-center distance between neighboring array elements must be as small as possible, preferably no more than 12 mm, which is the approximate minimum wavelength at 7.5 GHz in the breast-tissue phantoms used in the current study. Note that the Nyquist criterion dictates a sampling step as small as one-quarter of a wavelength under conditions of unattenuated far-field scattering [39]. However, the effective beamwidth of antennas operating in the lossy tissue environment is limited, which leads to relaxed spatial-sampling requirements.

Note that the choice of an optimal spatial sampling step is a compromise between the best use of the available effective spatial bandwidth of the data in Fourier (or *k*) space and avoiding over-sampling, which may lead to an ill-posed inversion problem [40,41,42]. Besides, over-sampling increases the acquisition time. The maximum recommended spatial sampling step is [39],

(1) $$\Delta \xi_{\max}=\frac{\lambda_{\mathrm{eff},\min}}{4\sin\alpha },\;\xi \equiv x,y,$$

where

(2) $$\lambda_{\mathrm{eff},\min}=\frac{2\pi }{k_{\xi }^{\max}},$$

is the minimum effective wavelength in the field distribution in the inspected region as determined from the data spatial spectral bandwidth $k_{\xi }^{\max}$. In addition, α is the maximum angle at which the target can be “seen” by the array. This angle is determined either by half of the antenna beamwidth or half of the angle subtended by the aperture, whichever is less. In tissue, the effective angle α is small because increasing it implies longer signal paths, which in turn result in weaker signals. The decrease of α leads to larger sampling steps $\Delta \xi_{max}$, ξ≡x,y, as recommended by Equations (1) and (2).

In summary, it is important to realize that denser sampling does not necessarily lead to improved imaging. The optimal choice of the spatial sampling step and pattern depends on many factors including the illumination beam and angles, the dielectric properties of the imaged tissue, as well as the maximum effective viewing angle of the receiving array. Bias-switched radio-sensor arrays will offer unprecedented flexibility in configuring the system optimally by the user who can control both the pattern and the sampling step by simply activating and deactivating array elements.

There is a great number of antennas designed to operate in direct contact with tissue (see, e.g., [5,18,21,43,44,45,46,47,48]). However, very few satisfy the design requirements above. Here, we have chosen to employ the antenna proposed in [49] as an initial design and to develop it further so that it meets all design specifications.

**Figure 2:**
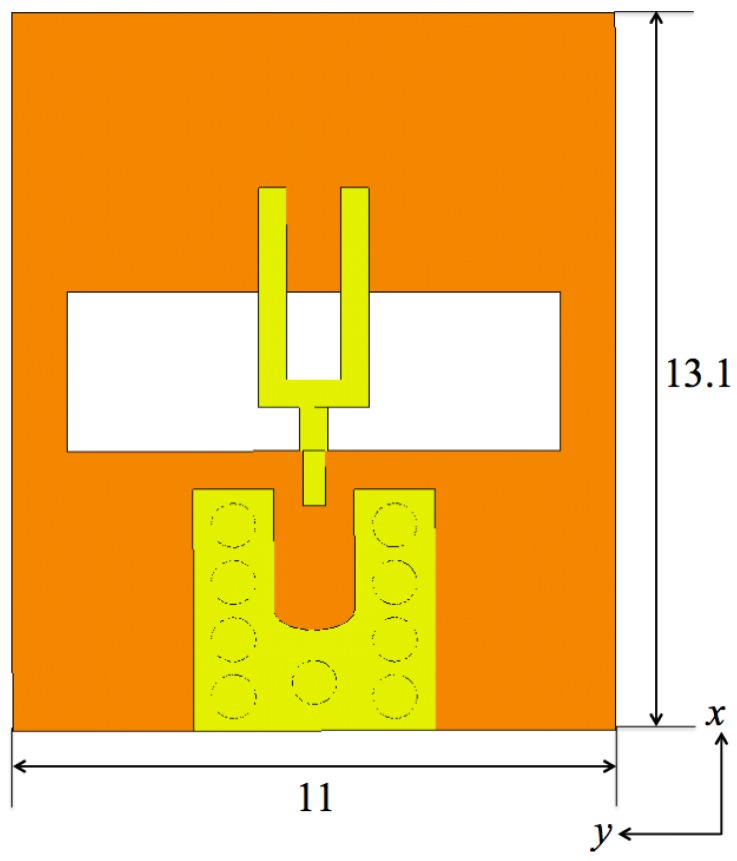
The layout of a single antenna. The fork and the slot dimensions as well as those of the feed pad are identical to those in [49]. The dimensions are in mm.

The proposed slot antenna is predominantly linearly polarized. It consists of two metalization layers on a 0.635 mm thick Rogers RT/Duroid 6010LM substrate with dielectric constant $\epsilon_{r}=10.2$ and loss tangent tanδ=0.002. Figure 2 shows the top (brown or dark gray) and the bottom (yellow or light gray) layers. A slot at the top layer is fed electromagnetically by a fork at the bottom layer. A ground pad at the bottom layer is connected to the top layer (the plane of the slot) with 9 vias. The ground of a surface-mount coaxial connector is to be soldered to the ground pad. The geometry and the sizes are identical to those in [49].

**Figure 3:**
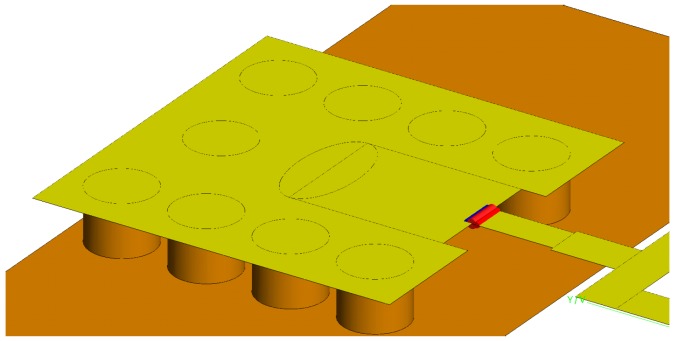
The edge port of the antenna when simulated in FEKO. The edge port is indicated by a red (dark-gray) cylinder at the base of the fork.

An edge-port feeding structure is used in FEKO [50] for the simulations of the antenna element. Among the various feeding options in FEKO, this type of port provides results tht agree best with the measurements. As shown in Figure 3, the edge-port implementation requires the metalization of the whole ground pad such that the edge port can be placed at the base of the fork. The yellow (or light-gray) part in Figure 3 shows the fork, the ground pad and the edge port. Thus, the simulated ground pad and the excitation in Figure 3 are different from the actual structure in Figure 2, which must be fed by a coaxial connector.

**Figure 4:**
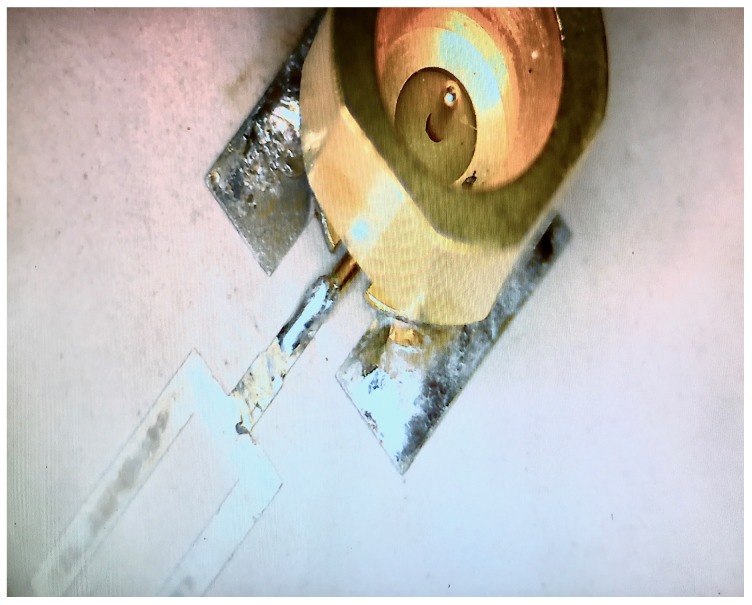
Mounted SMPM connector on the ground pad of the antenna element.

A single antenna prototype is fabricated and measured with a surface-mount sub-miniature push-on micro (SMPM) connector, the ground of which is soldered to the ground pad of the antenna as explained earlier. Figure 4 shows a photo of the SMPM coaxial connector soldered on top of the ground pad and the fork.

Further, unlike the prototype proposed in [49], which does not have backing layers or back-shielding, our antenna has been further developed to allow for full shielding from the back. It has been found that the antenna’s return loss is best if the metallization layer that contains the fork and the ground pad is backed by a 0.635 mm thick Rogers RT/Duroid 6010LM substrate. This is in turn backed by a 1.6-mm thick FR4 substrate with $\epsilon_{r}=4.6$ and tanδ=0.02, which in turn is shielded from the back with a layer of copper tape.

**Figure 5:**
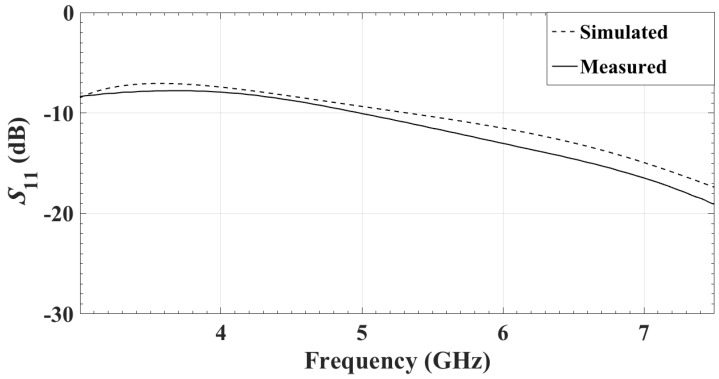
The simulated and the measured reflection coefficients of the single antenna.

**Figure 6:**
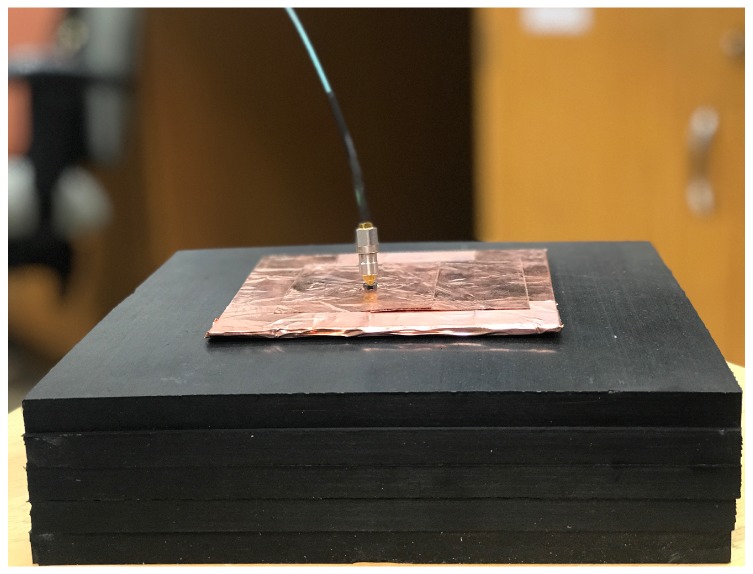
Measuring the reflection coefficient of the single antenna with a 5-cm thick carbon–rubber tissue phantom pressed against the antenna slot.

The simulated and the measured reflection coefficients (in dB) of the single antenna are shown in Figure 5. They match very well. The reflection coefficient is measured when the antenna is facing a 5-cm carbon–rubber tissue phantom, as shown in Figure 6. For the realistic thicknesses of the tissue (≥4 cm), $S_{11}$ is practically insensitive to changes in the thickness of the phantom.

**Figure 7:**
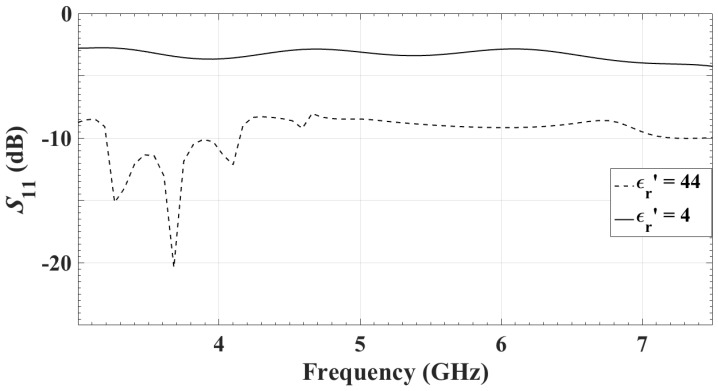
The reflection coefficient of the single antenna facing a medium of $\epsilon_{r}=4$ (representing fatty tissue) and $\epsilon_{r}=44$ (representing fibroglandular tissue).

Since the antennas are supposed to be used in direct contact with the skin, the $S_{11}$ dependence on the tissue dielectric properties is studied. Simulations suggest that changing the medium permittivity to that of fat, roughly $\epsilon_{r}\approx 4$, and fiberglandular, $\epsilon_{r}\approx 44$, results in changes of $S_{11}$, as shown in Figure 7.

### 2.3. Antenna Array

The array reported in [49] consists of 4 by 4 elements, which have been described in the previous section. The inter-element center-to-center spacing is 13.1 mm and 11 mm in the *x* and *y* directions, respectively.

**Figure 8:**
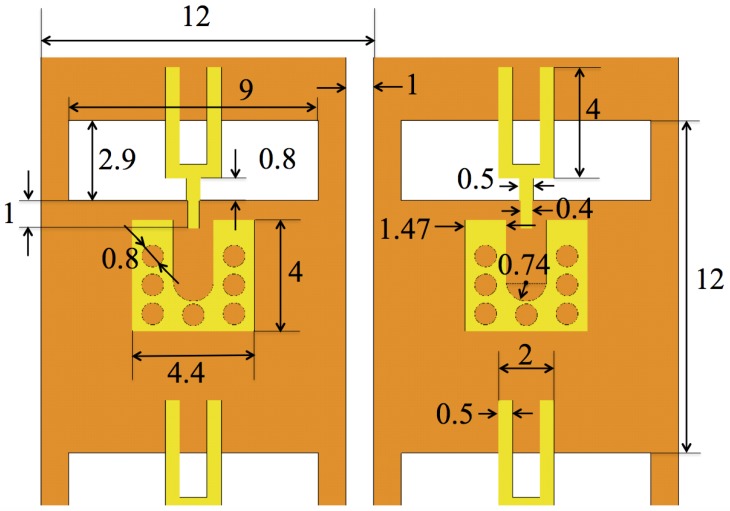
The layout of the antenna array (only four forks are shown). All dimensions are in mm.

The purpose here is to minimize the distance between the elements as much as possible to facilitate dense sampling. Moreover, we require that the distance between the array elements be the same in both the *x* and *y* directions. Since our system is bias-switched, the sensors are active one at a time. In other words, when any given sensor receives, all other sensors have their radios OFF. In this scenario, the mutual coupling between the antenna elements has little impact on the system dynamic range. This is one of the important advantages of using a bias-switched active sensor array. On the other hand, the proximity of other antenna elements does have a noticeable effect on the impedance match. The closer the antennas are, the worse the impedance match is of any given antenna element as compared to its impedance match when operating as a single element. Thus, a compromise is made between a minimal inter-element spacings and a good input impedance, with the inter-element spacings chosen as 12 mm by 12 mm. Figure 8 shows a portion of the array geometry with two neighboring elements. We should note that the element ground pad in our array is slightly changed compared to that in [49] and the one shown in Figure 2. The pad is now smaller and has only 7 vias, as shown in Figure 8.

**Figure 9:**
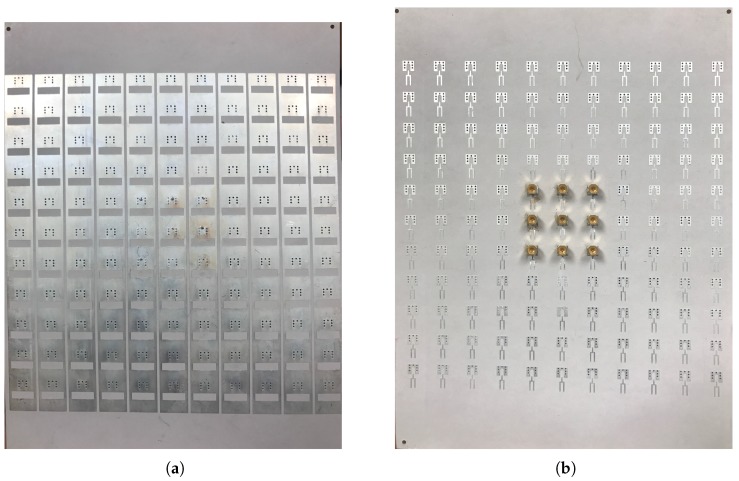
Photos of the fabricated antenna array consisting of 121 (11 by 11) elements: (**a**) top view of the metallization layer with the slots, which face the tissue phantom; and (**b**) bottom view of the metallization layer with the forks and the ground pads before mounting the backing dielectric layers and the metallic shielding. Nine of the center elements in a 3 by 3 configuration are soldered to SMPM connectors.

A prototype of the antenna array has been fabricated that consists of 121 (11 by 11) elements. Figure 9 shows the photos of the bottom and the top metallization layers of the PCB structure. The bottom view also shows 9 SMPM connectors soldered onto the central 9 elements of the array in a 3 by 3 configuration.

**Figure 10:**
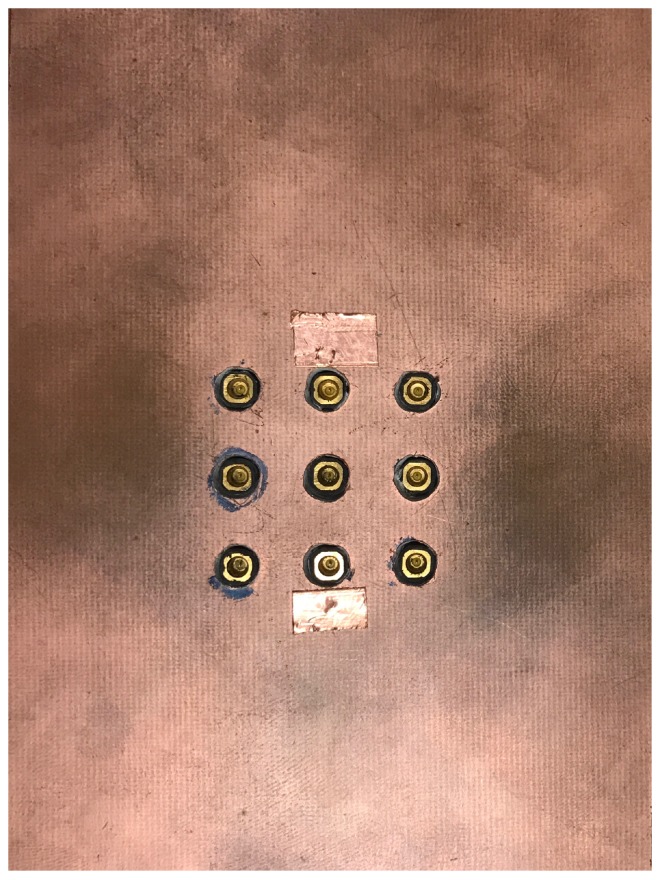
The bottom view of the designed array with shielding and SMPM connectors.

The backing layers have also been modified to account for the close proximity of the antenna elements and the modified ground pad. Various configurations have been designed and measured and the optimal case emerged as a double-layer structure. The first dielectric layer, directly backing the forks and the ground pads, is a 0.635 mm thick Rogers RT/Duroid 6010LM substrate ($\epsilon_{r}=10.2$, tanδ=0.002). This is in turn backed by a 3.2 mm thick FR4 substrate ($\epsilon_{r}=4.6$, tanδ=0.02). Finally, the whole structure is shielded with metal from the back. Figure 10 shows a photo of the bottom of the shielded array with the SMPM connectors attached to the central nine elements.

**Figure 11:**
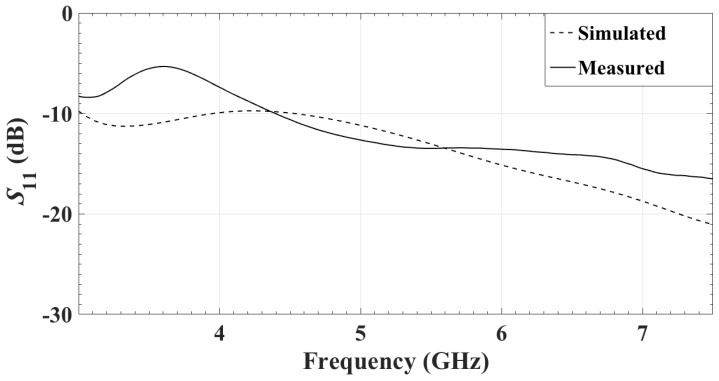
Reflection coefficient of the center element of the array.

The measured and simulated reflection coefficients of the center element of the array are compared in Figure 11. Differences are observed. They are due to the fact that the simulations are performed with a smaller array of 3 by 3 antennas, whereas the measured prototype consists of 11 by 11 antennas. Unfortunately, the simulation of the whole 121-element array has prohibitive computational demands. Another reason for the difference is that the simulation uses an edge-port excitation, whereas the actual prototype is excited through an SMPM connector. The simulation of an SMPM connector at each antenna element, even in the 3 by 3 array, results in very substantial increase in the computational time. There is also a difference between the tissue medium defined in FEKO and the actual tissue phantom used in the experiments. In the simulation, the tissue phantom is a 5-cm lossy dielectric layer, the constitutive parameters of which, $\epsilon_{r}=10.2$ and tanδ=0.5, are constant over the bandwidth. In addition, the layer is infinite in the *x* and *y* directions. On one the side, this layer interfaces with the antenna’s slot. On the other side, it interfaces a semi-infinite medium set as vacuum. In the measurements, the permittivity of the carbon–rubber tissue phantom is frequency-dependent (see Figure 1). Moreover, this tissue phantom, whose thickness is 5 cm, is finite in the *x* and *y* directions with its size being 20 cm by 20 cm. Thus, differences between the measured and the simulated results are expected.

### 2.4. Comparison Between a TEM Horn Antenna and the Proposed Antenna Element

We have compared our antenna array element with an UWB TEM horn antenna [45] designed to operate in direct contact with the carbon–rubber tissue phantoms. Note that the TEM horn is significantly larger than the proposed antenna element. Its aperture size is 31.2 mm by 22.6 mm and its length is 100 mm.

**Figure 12:**
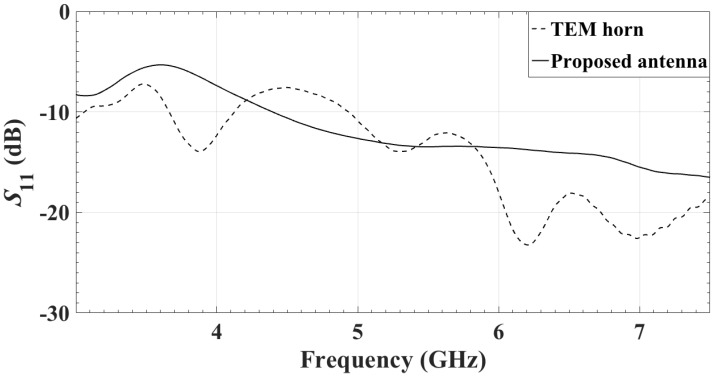
Measured reflection coefficients of the TEM horn antenna [45] and the center element of the proposed antenna array.

The measured reflection coefficients of the two antennas are compared in Figure 12. Both antennas are facing a 4-cm thick phantom. Despite being significantly smaller than the TEM horn, the proposed antenna has comparable return loss.

**Figure 13:**
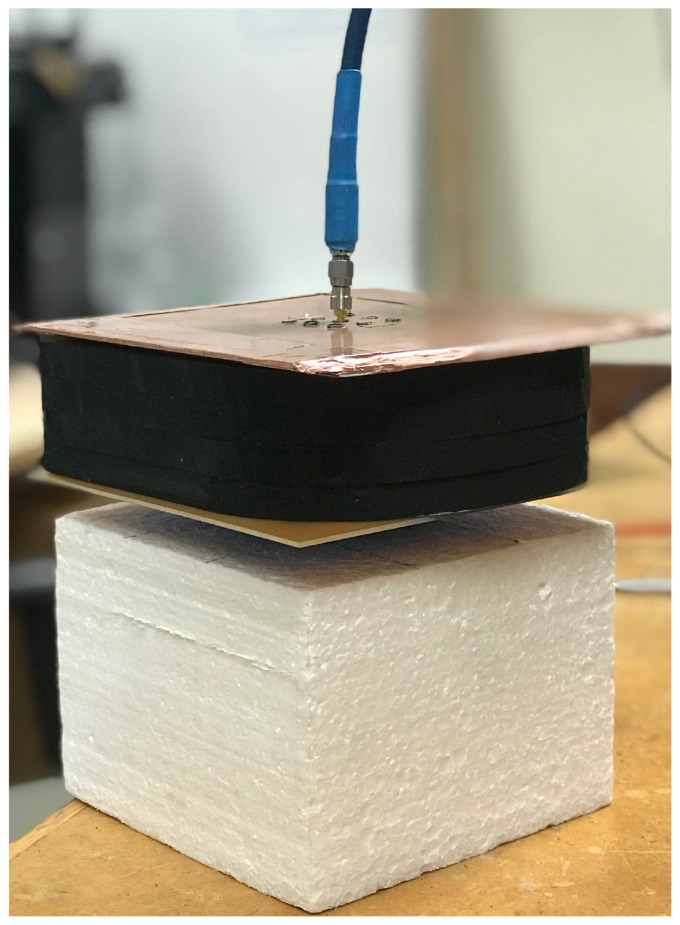
Measuring $S_{21}$ with a 4-cm thick tissue phantom.

**Figure 14:**
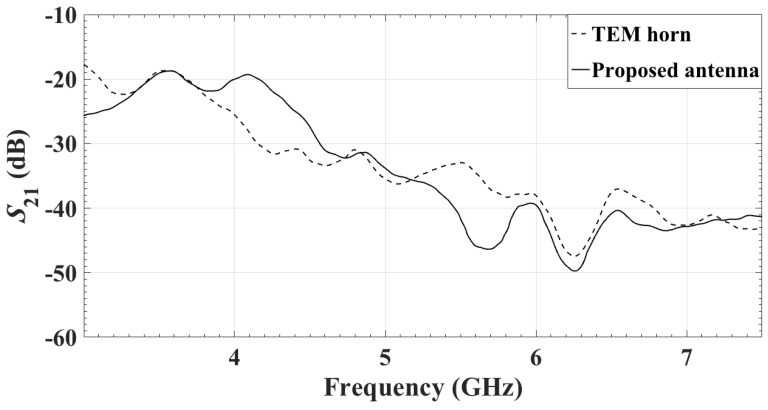
Measured transmission coefficients when Tx is in-house quadridge horn antenna [51] and Rx is either TEM horn antenna [45] or the center element of the proposed antenna array.

To compare the reception properties of the two antenna, an $S_{21}$ measurement is performed with an Agilent Technologies E8363B PNA Network Analyzer (10 MHz-40 GHz). Port 1 is attached to an in-house quadridge horn antenna [51], which acts as a transmitter. Port 2 is connected to either the center element of the antenna array or the UWB TEM horn. In both cases, the Rx antenna is co-polarized with the Tx one. A 4-cm thick carbon–rubber tissue phantom is inserted between the two antennas, as shown in Figure 13. The measured transmission coefficients are shown in Figure 14. It is evident that the reception capabilities of the two antennas are comparable.

Finally, to elucidate the advantages of the developed array element, a comparison is made in Table 2 with three other antenna elements used in microwave tissue imaging. These elements satisfy most of the design requirements for our application, i.e., they operate in the UWB, they can be fabricated on a PCB, they are smaller than 20 mm, and they do not employ resistive coating or absorbing materials. In addition, all of them have been integrated into arrays.

## 3. Mixer and LNA Parameter Measurements and their Integrations with the Antenna

### 3.1. Low-Noise Amplifier

The choice of the LNA is dependent on: (i) the bandwidth; (ii) the noise figure; (iii) the flatness of the gain across the bandwidth; and (iv) the size of the surface mount packaging. The PMA3-83LN+ LNA from *Mini-Circuits* is chosen as a suitable candidate. It is mounted on the evaluation board TB-830A+ [52]. Its noise figure is about 1.5 dB and its gain is 20.5 dB at 5 GHz. The gain varies from 18.5 dB to 20.5 dB within the bandwidth from 0.5 GHz to 8 GHz. The output power at the 1-dB compression point is 18.9 dBm.

### 3.2. Mixer

**Figure 15:**
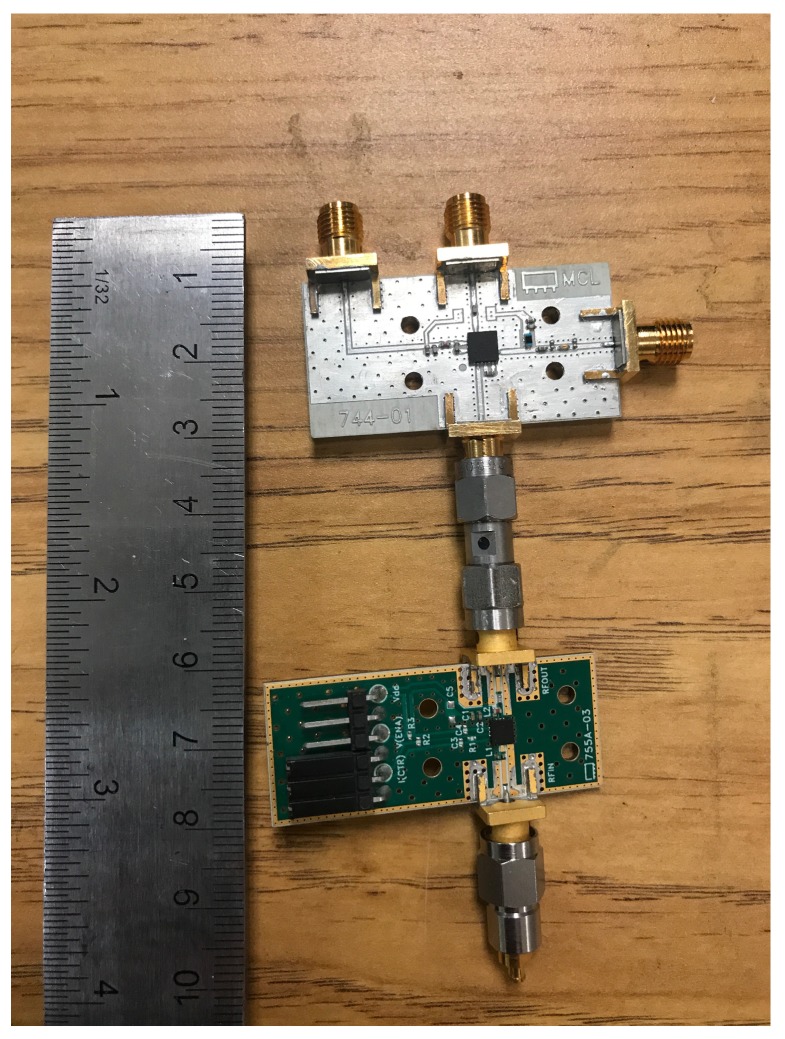
The evaluation boards of the selected mixer (white) and the LNA (green). The LNA and the mixer are connected together with SMA male to male adapter, and the LNA is connected to SMA to SMPM adapter to make it possible to integrate with the antenna array.

The mixer is chosen based on the following requirements: (i) RF bandwidth approximately from 3 GHz to 8 GHz; (ii) IF bandwidth of 30 MHz or less; (iii) low local oscillator (LO) drive power; and (iv) small surface mount packaging. These requirements are satisfied by the MDA4-752H+ mixer mounted on the evaluation board TB-771+ [53] from *Mini-Circuits* (the evaluation boards for the mixer and the LNA are shown in Figure 15). This active mixer has a gain between 4 dB and 9 dB in the bandwidth from 2.2 GHz to 7.5 GHz. It also has a low-noise IF amplifier to enable positive conversion gain. The noise figure is about 10 dB and the input 1-dB compression point is 9 dBm.

The mixer is measured at different RF frequencies for an IF of 30 MHz. The Agilent Technologies E8363B PNA Network Analyzer (10 MHz-40 GHz) is used as the RF power generator. The HP 8671B Synthesized CW Generator is used as an LO. The IF power is then measured with the Tektronix RSA 6114A Real-Time Spectrum Analyzer. Some representative results are shown in Table 3.

The radio, which consists of the mixer and the LNA, is measured at various RF frequencies for an IF of 30 MHz. Port 1 of the Agilent Technologies E8363B PNA Network Analyzer (10 MHz-40 GHz) is connected to the input of the LNA whose output is attached to the RF port of the mixer. The HP 8671B Synthesized CW Generator is used as an LO. The IF power is measured with the Tektronix RSA 6114A Real-Time Spectrum Analyzer.

The input power for the LNA is set to −30 dBm for all three frequencies. These representative results are shown in Table 4. In the whole bandwidth from 3 GHz to 7.5 GHz, the system gain varies between 19.9 dB and 30.2 dB.

### 3.3. Integrating the LNA and the Mixer with the Antenna

**Figure 16:**
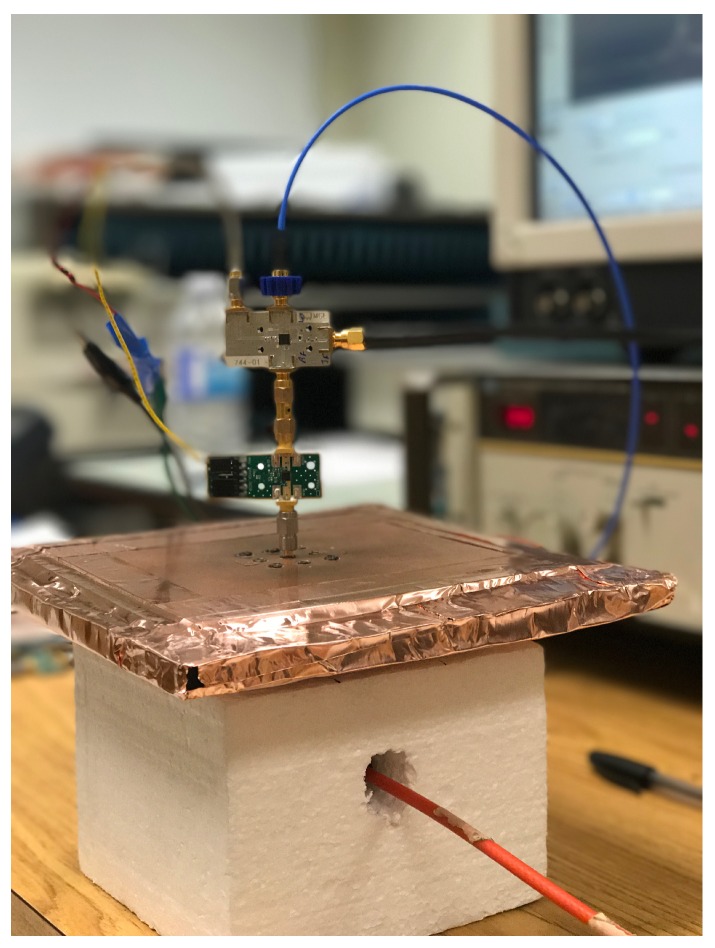
The radio-sensor prototype consisting of the antenna, the LNA and the mixer.

For testing purposes, the LNA and the mixer are connected to the central element of the array. An in-house quadridge horn antenna [51] is used as the transmitting element. The quadridge horn is designed for best impedance match when it operates in direct contact with the carbon–rubber tissue phantoms, the permittivity of which is shown in Figure 1. We have constructed a Styrofoam box as shown in Figure 16 to mount the transmitting antenna and the radio sensor conveniently. The transmitting antenna is placed in the Styrofoam box facing up. The tissue phantom rests on top of the Styrofoam box. Its thickness is 1 cm and its lateral size is 20 cm by 20 cm. The antenna-array structure rests on top of the tissue phantom with the slot facing the tissue phantom. The whole structure is coated with copper tape from the top and the sides. The central antenna element is connected to the LNA board, which in turn is connected to the mixer board.

## 4. Results

### 4.1. Dynamic Range of the Proposed Active Sensor

The mixer and the LNA are biased with 5 V DC power supply. The IF output of the mixer is connected to Tektronix RSA 6114A Real-Time Spectrum Analyzer (9 kHz to 14 GHz). The LO power is supplied with HP 8671B Synthesized CW Generator (2 GHz to 18 GHz).

The amount of the power that can be fed to the transmitting antenna depends on the saturation power of the LNA and the 1-dB compression point of the mixer. In our case, the maximum received power is limited by the 1-dB compression point of the mixer, which is 9 dBm at the RF input. Thus, in this prototype, the dynamic range is limited from above by the mixer. From below, the dynamic range is limited by the output IF power that the radio would produce when the bias of the LNA and the mixer is OFF. As is shown shortly, this IF power is undetectable since it is well below the noise of the spectrum analyzer. It is therefore the noise of the spectrum analyzer that limits the dynamic range from below.

The power fed to the transmitting antenna is set at 21 dBm, which leads to an input RF power at the mixer close to the maximum. The power levels at the IF output of the mixer are then measured with the spectrum analyzer when the bias of the LNA and the mixer is ON and OFF. Note that the transmitting antenna radiates continuously, regardless of whether the bias of the sensor is ON or OFF. Representative results are shown here with the following settings:

- RF frequency at 3.03 GHz, 5.03 GHz, and 7.5 GHz;
- LO frequency at 3 GHz, 5 GHz, and 7.47 GHz, respectively (so that the IF is at 30 MHz); and
- LO power at 0 dBm (as per the recommendation in the mixer datasheet).

**Figure 17:**
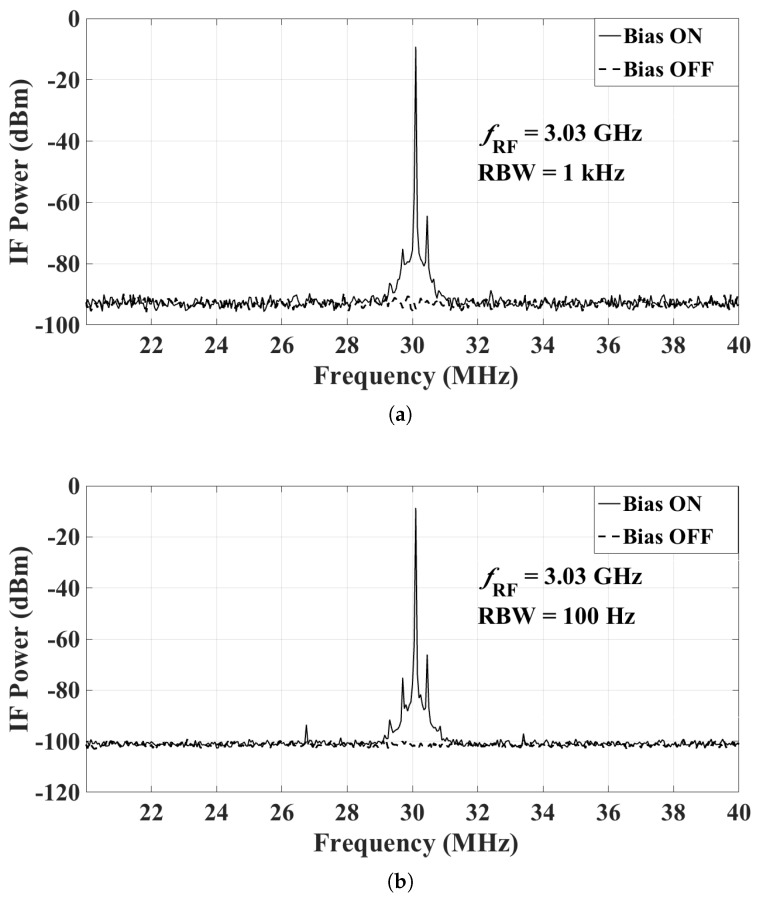
The IF output of the mixer at RF of 3.03 GHz with a 20-dB attenuator attached at the input of the spectrum analyzer: (**a**) RBW = 1 kHz; and (**b**) RBW = 100 Hz.

**Figure 18:**
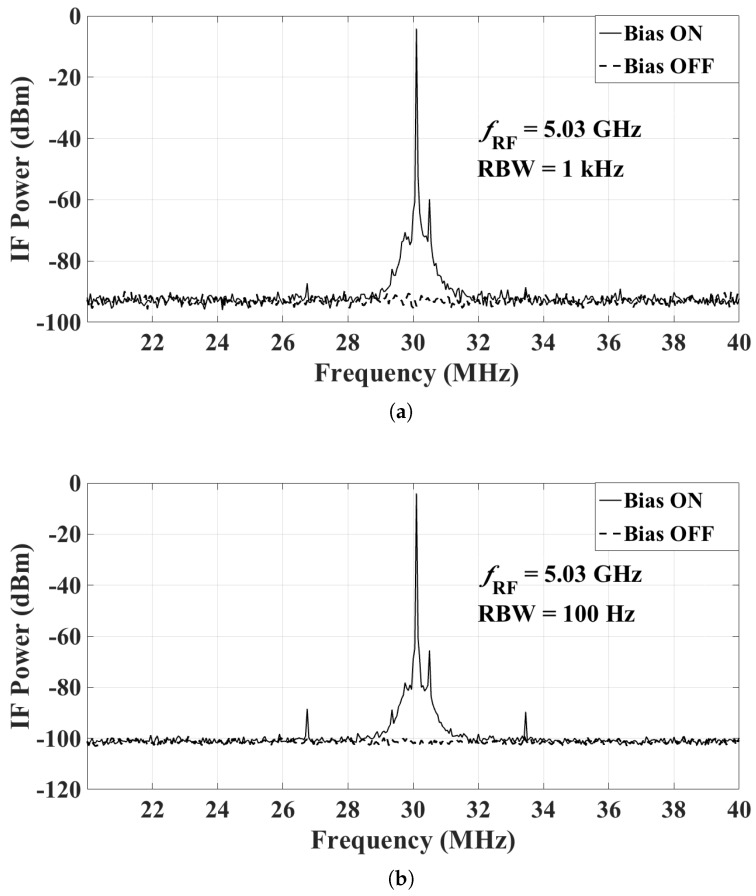
The IF output of the mixer at RF of 5.03 GHz with a 20-dB attenuator attached at the input of the spectrum analyzer: (**a**) RBW = 1 kHz; and (**b**) RBW = 100 Hz.

**Figure 19:**
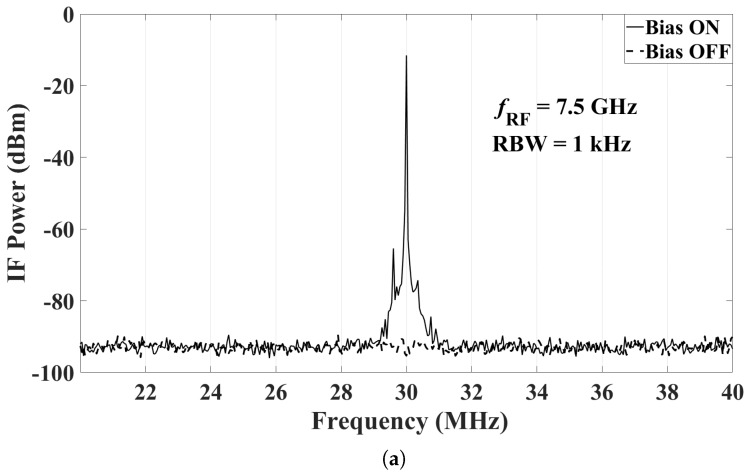

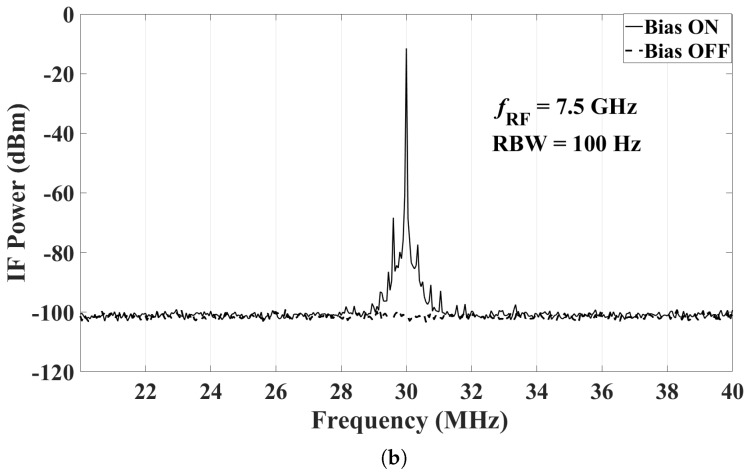
The IF output of the mixer at RF of 7.5 GHz with a 20-dB attenuator attached at the input of the spectrum analyzer: (**a**) RBW = 1 kHz; and (**b**) RBW = 100 Hz.

**Figure 20:**
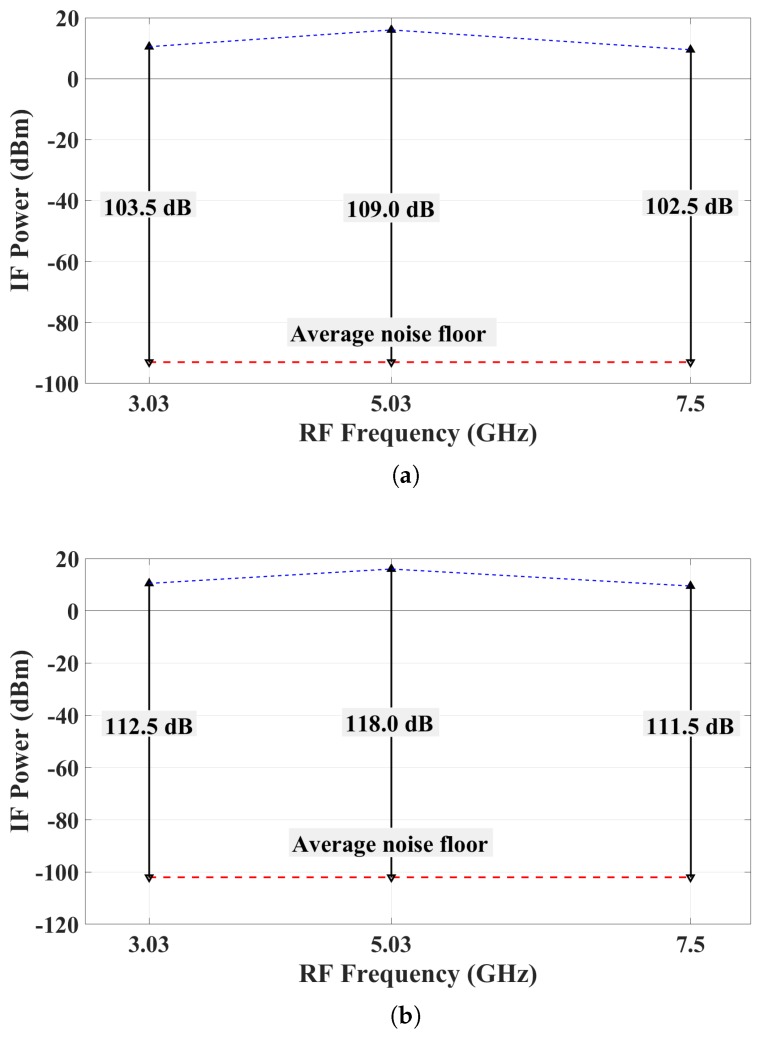
The dynamic range of the proposed system at different frequencies: (**a**) RBW = 1 kHz; and (**b**) RBW = 100 Hz.

The IF output is measured with two different resolution bandwidths (RBW), 1 kHz and 100 Hz. A 20-dB attenuator is attached to the input port of the spectrum analyzer to protect the instrument. Figure 17a, Figure 18a and Figure 19a show the IF output with the RBW set to 1 kHz, when $f_{\mathrm{RF}}$ is 3.03 GHz, 5.03 GHz and 7.5 GHz, respectively. Figure 17b, Figure 18b and Figure 19b show the IF output with the RBW set to 100 Hz. In these figures, the IF signal power in the ON state has weak side band spectral lines. This is because the mixer output is not filtered. The narrow-band IF filtering is to be performed after the common IF port, which collects the signals from all sensors. We reiterate that a signal is effectively generated from one sensor at a time, the sensor whose bias is ON. At $f_{\mathrm{RF}}=5.03$ GHz, the IF power level with the bias ON is −4.0 dBm at 30 MHz, which corresponds to an actual received power of 16 dBm after taking into account the attenuator. From these results, it is clear that when the bias of the radio is OFF, its measured IF output is undetectable, i.e., it is within the noise of the spectrum analyzer. The average noise floor of the system is about −93 dBm and −102 dBm for RBW of 1 kHz and 100 Hz, respectively. Thus, the dynamic range of the whole system at $f_{\mathrm{RF}}=5.03$ GHz is estimated to be 109 dB and 118 dB for RBW of 1 kHz and 100 Hz, respectively. The dynamic range estimates for all three measured frequencies are summarized in Figure 20.

### 4.2. Comparison with Conventional RF-Switch System

**Figure 21:**
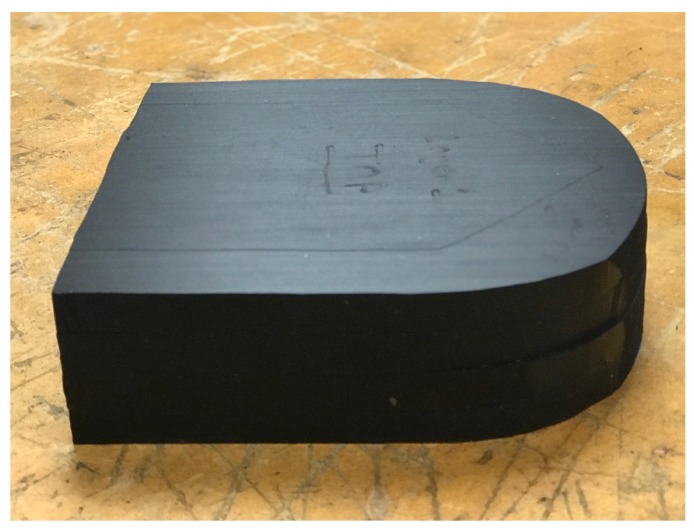
4-cm thick tissue phantom.

Next, we compare the received IF power from the system in two different system arrangements. System 1 is as explained in the previous section. More specifically, RF power is transmitted by the quad-ridge horn antenna [51] into a 4-cm thick tissue phantom (see Figure 21). The power fed to the Tx horn is 15 dBm. It is then received by the central element of the proposed active array, which is positioned on the opposing side of the phantom and aligned with the boresight of the Tx horn. The IF power is received by the spectrum analyzer.

System 2 uses an RF switch. The Tx setup is the same as in the first configuration. On the Rx side, however, the mixer and the LNA are detached from the antenna element. The antenna element is connected directly to the input of an RF switch (Advantest R3970 multi-port test adapter). The output of the RF switch is connected to the LNA and the mixer, which in turn is connected to the spectrum analyzer.

The received IF power levels in both systems are shown in Table 5. It is evident that the received IF power in System 1 (without RF switch) is significantly larger. This is because System 2 suffers from higher loss due to the RF switch, an additional coaxial cable and two additional connectors. To illustrate the loss due to the above components in System 2, Table 6 provides the measured losses of the RF switch along with the corresponding values found in the Users Manual [54]. Note that the measured loss in Table 6 includes not only the loss in the signal paths of the RF switch, but also that due to the cable and connectors.

## 5. Discussion

This manuscript proposes a new approach to designing microwave-imaging hardware; it does *not* propose a new imaging method. In particular, it proposes a new active sensor, which incorporates both an antenna and a radio front end. The sensor is controlled to assume an ON and an OFF state by simply switching the radio bias on and off. The goal of developing a bias-switched active radio sensor, is to enable the multiplexing of hundreds to thousands of sensors in an array. Such large-scale multiplexing is practical only if the output of each sensor is at a low narrow-band frequency range (in the low *mega-hertz* range), so that the signal transmission and distribution networks are simple, low-loss and low-distortion. Thereby, the sensor output must be at IF. Such technological development would be beneficial to microwave imaging in general but tissue imaging can benefit the most as it suffers from poor data quality due to high loss, high noise, uncertainty, and insufficient spatial sampling. Current RF-switched technology is limited to several tens of antennas, resulting in insufficient spatial sampling. Antenna scanning can provide fine sampling but is prohibitively slow and suffers from positioning uncertainties. The proposed active sensor array offers a solution where scanning is avoided, yet thousands of spatial samples can be gathered within seconds through electronic switching of the bias network of the array.

The active radio sensor investigated here is to be used in an UWB bias-switched array for microwave imaging of the breast. This leads to the antenna design requirements, such as UWB bandwidth (preferably between 3 GHz and 7 GHz or more), small size (≤12 mm on a side), light weight, and PCB technology. We have demonstrated that the sensor can be used to easily build a large planar array on a PCB (11×11 elements providing an acquisition surface of 144×144 mm${}^{2}$). The sensor is sufficiently small to be used in building arrays of any desired shape and size.

We emphasize that the proposed array is receiving, i.e., it does not transmit. The illumination of the object under test (OUT) can be realized by any desired antenna or an antenna array. To validate the performance of the proposed bias-switched radio sensor, we use an in-house UWB TEM horn antenna here [51]. It is also possible to modify this array to include not only receiving elements but also transmitting elements thereby enabling single-sided (or back-scattering) measurements.

It is worth mentioning that a prototype of the radio sensor was also built based on a passive mixer. The advantages of the passive mixer are in its broader bandwidth and a higher 1-dB compression point. Its disadvantage is in the conversion loss, which requires an additional IF amplifier, which would make the integration and the miniaturization of the sensor more difficult. Without an additional IF amplifier, the sensor based on a passive mixer has a lower dynamic range.

## 6. Conclusions

An active radio sensor for microwave tissue imaging is developed and measured. This sensor integrates a printed slot antenna with a chip LNA and a mixer, with its output signal being at an IF of 30 MHz. The radio sensor is intended to serve as an element in large bias-switched tissue-imaging arrays. The measured dynamic range of the bias-switched radio sensor is 109 dB and 118 dB with IF resolution bandwidths of 1 kHz and 100 Hz, respectively. Future work includes: (i) further miniaturization by the integration of the chip LNA with the slot antenna on a common PCB; and (ii) integration with the wireless-transmission module supplying the local-oscillator (LO) signal. The first generation of the bias-switched sensor array is under development, where the LNA is integrated with the antenna on the same printed circuit board (PCB), forming an active antenna. Further, the active antenna array is to be integrated with a matching array of mixers on a multi-layer PCB. In the second generation, we envision integrating the whole radio sensor in a single chip. The final goal is a bias-switched imaging array of 400 radio sensors in a 20 by 20 configuration, which will allow for the fast acquisition of both magnitude and phase in the frequency bandwidth from 3 GHz to 7.5 GHz over an area of 24 cm by 24 cm.

## Figures and Tables

**Table 1:** Comparison between the proposed active bias-switched array and other microwave imaging systems.

	Antenna Scanning	RF-Switched Array	MST Array	Bias-Switched Array
Input/Output Frequency	RF/RF	RF/RF	RF/RF	RF/IF
Number of Multiplexed Ports	9 [21]	60 [18]	1024 [24]	Hundreds/Thousands
Acquisition Time	Slow	Fast	Fast	Fast
Positioning Uncetianties	High	Low	Low	Low
Interconnect Complexity	Low	High	Low	Low
Interconnect Losses and Distortion	High	High	Very High	Low

**Table 2:** Comparison with relevant UWB antenna arrays for microwave tissue imaging.

	The Largest Inter-Element Spacing (mm)	Bandwidth (GHz)	Back Shielding	Number of Elements
Antenna Reported in [18]	>14	3.8–10	Yes	60
Antenna Reported in [49]	13.1	2.6–16	No	16
The Proposed Antenna Array	12	2.6–9	Yes	121

**Table 3:** IF Measurements for MDA4-752H+.

	RF	LO	IF	Mixer Gain (dB)
Power (dBm)	−5	0	4.6	9.6
Frequency (GHz)	3.03	3	0.03		
Power (dBm)	−5	0	1.6	6.6
Frequency (GHz)	5.03	5	0.03		
Power (dBm)	−5	0	−3.2	1.8
Frequency (GHz)	7.5	7.47	0.03		

**Table 4:** IF measurements of the LNA and the mixer.

	RF	LO	IF	System Gain (dB)
Power (dBm)	−30	0	0.2	30.2
Frequency (GHz)	3.03	3	0.03		
Power (dBm)	−30	0	−3.4	26.6
Frequency (GHz)	5.03	5	0.03		
Frequency (GHz)	7.5	7.47	0.03	

**Table 5:** Comparison between the received IF power in two systems.

	System 1 (dBm)	System 2 (dBm)	Difference (dB)
$f_{\mathrm{RF}}$ = 3.03 GHz	−19	−25	6
$f_{\mathrm{RF}}$ = 5.03 GHz	−27	−35	8
$f_{\mathrm{RF}}$ = 7.5 GHz	−34	−41	7

**Table 6:** Loss due to RF-switch: Measured value and value from users manual.

	Measured (dB)	From Users Manual [54] (dB)
3.03 GHz	6	6 or less
5.03 GHz	8	7 or less
7.5 GHz	7	8.5 or less

## References

[1] Nikolova N.K. (2011). Microwave imaging for breast cancer. IEEE Microw. Mag..

[2] Ley S., Sachs J., Helbig M. MNP enhanced microwave breast cancer imaging based on ultra-wideband pseudo-noise sensing. Proceedings of the 11th European Conference on Antennas and Propagation (EUCAP).

[3] Conceição R.C., Mohr J.J., O’Halloran M. (2016). An Introduction to Microwave Imaging for Breast Cancer Detection.

[4] Kwon S., Lee S. (2016). Recent advances in microwave imaging for breast cancer detection. Int. J. Biomed. Imaging.

[5] Bucci O.M., Bellizzi G., Borgia A., Costanzo S., Crocco L., Massa G.D., Scapaticci R. (2017). Experimental framework for magnetic nanoparticles enhanced breast cancer microwave imaging. IEEE Access.

[6] Cheng G.G., Zhu Y., Grzesik J. Microwave imaging of biological bone tissue. Proceedings of the USNC-URSI Radio Science Meeting (Joint with AP-S Symposium).

[7] Meaney P.M., Goodwin D., Golnabi A., Pallone M., Geimer S., Paulsen K.D. 3D microwave bone imaging. Proceedings of the 6th European Conference on Antennas and Propagation (EUCAP).

[8] Ireland D., Bialkowski K., Abbosh A. (2013). Microwave imaging for brain stroke detection using Born iterative method. IET Microw. Antennas Propag..

[9] Tournier P.H., Bonazzoli M., Dolean V., Rapetti F., Hecht F., Nataf F., Aliferis I., El-Kanfoud I., Migliaccio C., de Buhan M., Darbas M., Semenov S., Pichot C. (2017). Numerical modeling and high-speed parallel computing: new perspectives on tomographic microwave imaging for brain stroke detection and monitoring. IEEE Antennas Propag. Mag..

[10] Dilman İ., Yıldırım U., Coşğun S., Doğu S., Çayören M., Akduman I. Feasibility of brain stroke imaging with microwaves. Proceedings of the IEEE Asia-Pacific Conference on Applied Electromagnetics (APACE).

[11] Zamani A., Rezaeieh S.A., Abbosh A.M. (2015). Lung cancer detection using frequency-domain microwave imaging. Electron. Lett..

[12] Babarinde O.J., Jamlos M.F., Soh P.J., Schreurs D.M.M.P., Beyer A. Microwave imaging technique for lung tumour detection. Proceedings of the German Microwave Conference (GeMiC).

[13] Nikolova N.K. (2014). Microwave biomedical imaging. Wiley Encyclopedia of Electrical and Electronics Engineering.

[14] Lazebnik M., McCartney L., Popovic D., Watkins C.B., Lindstrom M.J., Harter J., Sewall S., Magliocco A., Booske J.H., Okoniewski M., Hagness S.C. (2007). A large-scale study of the ultrawideband microwave dielectric properties of normal breast tissue obtained from reduction surgeries. Phys. Med. Biol..

[15] Lazebnik M., Popovic D., McCartney L., Watkins C.B., Lindstrom M.J., Harter J., Sewall S., Ogilvie T., Magliocco A., Breslin T.M. (2007). A large-scale study of the ultrawideband microwave dielectric properties of normal, benign and malignant breast tissues obtained from cancer surgeries. Phys. Med. Biol..

[16] Halter R.J., Zhou T., Meaney P.M., Hartov A., Barth Jr R.J., Rosenkranz K.M., Wells W.A., Kogel C.A., Borsic A., Rizzo E.J. (2009). The correlation of in vivo and ex vivo tissue dielectric properties to validate electromagnetic breast imaging: initial clinical experience. Physiol. Meas..

[17] Sugitani T., Kubota S.i., Kuroki S.i., Sogo K., Arihiro K., Okada M., Kadoya T., Hide M., Oda M., Kikkawa T. (2014). Complex permittivities of breast tumor tissues obtained from cancer surgeries. Appl. Phys. Lett..

[18] Klemm M., Gibbins D., Leendertz J., Horseman T., Preece A., Benjamin R., Craddock I. Development and testing of a 60-element UWB conformal array for breast cancer imaging. Proceedings of the 5th European Conference on Antennas and Propagation (EUCAP).

[19] Song H., Sasada S., Kadoya T., Okada M., Arihiro K., Xiao X., Kikkawa T. (2017). Detectability of breast tumor by a hand-held impulse-radar detector: performance evaluation and pilot clinical study. Sci. Rep..

[20] Amineh R.K., Ravan M., Khalatpour A., Nikolova N.K. (2011). Three-dimensional near-field microwave holography using reflected and transmitted signals. IEEE Trans. Antennas Propag..

[21] Amineh R.K., McCombe J.J., Khalatpour A., Nikolova N.K. (2015). Microwave holography using point-spread functions measured with calibration objects. IEEE Trans. Instrum. Meas..

[22] Ostadrahimi M., Asefi M., LoVetri J., Bridges G.E., Shafai L. An MST-based microwave tomography system using homodyne receiver. Proceedings of the 2013 IEEE Antennas and Propagation Society International Symposium (APSURSI).

[23] Crocker D.A., Donnell K.M. (2015). Application of electrically invisible antennas to the modulated scatterer technique. IEEE Trans. Instrum. Meas..

[24] Joisel A., Mallorqui J., Broquetas A., Geffrin J., Joachimowicz N., Lossera M., Joire L., Bolomey J.C. Microwave imaging techniques for biomedical applications. Proceedings of the 16th IEEE on Instrumentation and Measurement Technology Conference IMTC/99.

[25] Bolomey J.C., Gardiol F.E. (2001). Engineering Applications of the Modulated Scatterer Technique.

[26] Abou-Khousa M.A., Ghasr M.T., Kharkovsky S., Pommerenke D., Zoughi R. (2011). Modulated elliptical slot antenna for electric field mapping and microwave imaging. IEEE Trans. Antennas Propag..

[27] Sturley K.R. (1953). Radio Receiver Design.

[28] Agilent AN 1275, Automatic Frequency Settling Time Measurements Speeds Time-to-Market for RF Design: Application Note. http://literature.cdn.keysight.com/litweb/pdf/5964-4335E.pdf.

[29] Guan X., Hashemi H., Hajimiri A. (2004). A fully integrated 24-GHz eight-element phased-array receiver in silicon. IEEE J. Solid-State Circuits.

[30] Tajik D., Foroutan F., Shumakov D.S., Pitcher A.D., Nikolova N.K. (2017). Real-time microwave imaging of a compressed breast phantom with planar scanning. IEEE J. Electromagn. RF Microw. Med. Biol..

[31] Beaverstone A.S., Nikolova N.K. Modeling and design of a switched transceiver array for tissue imaging. Proceedings of the IEEE MTT-S International Conference on Numerical Electromagnetic and Multiphysics Modeling and Optimization (NEMO).

[32] Beaverstone A.S., Nikolova N.K. Switched sensor array for near-field microwave imaging of tissue. Proceedings of the IEEE International Symposium on Antennas and Propagation & USNC/URSI National Radio Science Meeting.

[33] ACR BI-RADS^®^ ATLAS-MAMMOGRAPHY. https://www.acr.org/-/media/ACR/Files/RADS/BI-RADS/Mammography-Reporting.pdf.

[34] University of Wisconsin-Madison Numerical Breast Phantom Repository. https://uwcem.ece.wisc.edu/phantomRepository.html.

[35] Trehan A. (2009). Numerical and Physical Models for Microwave Breast Imaging. Master’s Thesis.

[36] Moll J., Wcõrtge D., Krozer V., Santorelli A., Popović M., Bazrafshan B., Hübner F., Vogl T.J., Nikolova N. Quality control of carbon-rubber tissue phantoms: Comparative MRI, CT, X-ray and UWB microwave measurements. Proceedings of the 11th European Conference on Antennas and Propagation (EUCAP).

[37] Lazebnik M., Okoniewski M., Booske J.H., Hagness S.C. (2007). Highly accurate Debye models for normal and malignant breast tissue dielectric properties at microwave frequencies. IEEE Microw. Wirel. Compon. Lett..

[38] Baskharoun Y., Trehan A., Nikolova N.K., Noseworthy M.D. Physical phantoms for microwave imaging of the breast. Proceedings of the 2012 IEEE Topical Conference on Biomedical Wireless Technologies, Networks, and Sensing Systems (BioWireleSS).

[39] Nikolova N.K. (2017). Introduction to Microwave Imaging.

[40] Bucci O., Franceschetti G. (1987). On the spatial bandwidth of scattered fields. IEEE Trans. Antennas Propag..

[41] Bucci O., Isernia T. (1997). Electromagnetic inverse scattering: retrievable information and measurement strategies. Radio Sci..

[42] Bucci O.M., Crocco L., Scapaticci R., Bellizzi G. (2016). On the design of phased arrays for medical applications. Proc. IEEE.

[43] Klemm M., Craddock I.J., Leendertz J.A., Preece A., Benjamin R. (2009). Radar-based breast cancer detection using a hemispherical antenna array; experimental results. IEEE Trans. Antennas Propag..

[44] Gibbins D., Klemm M., Craddock I.J., Leendertz J.A., Preece A., Benjamin R. (2010). A comparison of a wide-slot and a stacked patch antenna for the purpose of breast cancer detection. IEEE Trans. Antennas Propag..

[45] Amineh R.K., Trehan A., Nikolova N.K. (2009). TEM horn antenna for ultra-wide band microwave breast imaging. Prog. Electromagn. Res. B.

[46] Kanj H., Popovic M. (2005). Miniaturized microstrip-fed “Dark Eyes” antenna for near-field microwave sensing. IEEE Antennas Wirel. Propag. Lett..

[47] Islam M.A., Kiourti A., Volakis J.L. A novel body-worn RF sensor for deep tissue imaging. Proceedings of the 2015 IEEE MTT-S 2015 International Microwave Workshop Series on RF and Wireless Technologies for Biomedical and Healthcare Applications (IMWS-BIO).

[48] Aguilar S.M., Al-Joumayly M.A., Burfeindt M.J., Behdad N., Hagness S.C. (2014). Multiband miniaturized patch antennas for a compact, shielded microwave breast imaging array. IEEE Trans. Antennas Propag..

[49] Sugitani T., Kubota S., Toya A., Kikkawa T. (2012). A compact 4X4 planar UWB antenna array for 3-D breast cancer detection. IEEE Antennas Propag. Soc. Int. Symp..

[50] EM Software and Systems. https://altairhyperworks.com/product/FEKO.

[51] Dadash S., Moussakhani K., McCombe J. (2014). Quad-Ridge Horn Antenna for Tissue Measurements.

[52] TB-830A+ Evaluation Board. https://www.minicircuits.com/WebStore/dashboard.html?model=TB-830A%2B.

[53] TB-771+ Evaluation Board. https://www.minicircuits.com/WebStore/dashboard.html?model=MDA4-752H%2B.

[54] R3969/R3970-Advantest. https://www.advantest.com/documents/11348/146687/pdf_mn_ER3969_OPERATING_MANUAL.pdf.

